# Choreographing root architecture and rhizosphere interactions through synthetic biology

**DOI:** 10.1038/s41467-024-45272-5

**Published:** 2024-02-14

**Authors:** Carin J. Ragland, Kevin Y. Shih, José R. Dinneny

**Affiliations:** Department of Biology, Stanford University, Stanford, CA 94305 USA

**Keywords:** Molecular engineering in plants, Plant physiology, Rhizobial symbiosis, Synthetic biology

## Abstract

Climate change is driving extreme changes to the environment, posing substantial threats to global food security and bioenergy. Given the direct role of plant roots in mediating plant-environment interactions, engineering the form and function of root systems and their associated microbiota may mitigate these effects. Synthetic genetic circuits have enabled sophisticated control of gene expression in microbial systems for years and a surge of advances has heralded the extension of this approach to multicellular plant species. Targeting these tools to affect root structure, exudation, and microbe activity on root surfaces provide multiple strategies for the advancement of climate-ready crops.

## Abstract (web summary)

Engineering the form and function of root systems and their associated microbiota could provide a means to mitigate adverse climate-driven effects. Here, the authors review the recent developments in plant and rhizobacterial synthetic biology and highlight engineering targets for applications in root systems and rhizosphere.

## Introduction

In plants, the leap from genetic manipulation to genetic engineering will require knowledge of endogenous regulatory mechanisms and the establishment of precise molecular tools to modulate gene expression across time, space, and magnitude. Developing such control over gene regulation will unlock the potential of our crops to become more resilient in extreme weather and to rebalance carbon ratios between the atmosphere and global soils–the predominant terrestrial repository of carbon and de facto plant growth medium. Nonetheless, the absence of advanced genetic tools capable of reliably and predictably altering plant structure and functionality has impeded progress in this innovative field. In contrast, bacterial engineering benefits from a suite of well-characterized tools for both design and genetic manipulation. Naturally isolated root colonizing bacteria, known as rhizobacteria, can influence host plant immunity and development through the synthesis of plant hormones, and provide key services such as pathogen biocontrol and nutrient synthesis/solubilization. These traits can be transferred, tailored, and enhanced through synthetic biology approaches to create novel strains for improving crop resilience and soil carbon storage.

As they are the primary biological interface with soil, roots are a clear engineering target to sequester atmospheric carbon and fortify crops against stress. The functionality of roots is governed by the architectural layout of the root branches that dictates the extent of soil explored, the tissue types that express transporters to facilitate nutrient and water absorption, and the complex milieu of metabolites exuded that mediate interactions with soil microorganisms^1^. The integration of abiotic and biotic triggers on developmental and physiological responses allow for plasticity in root function that can manifest in diverse topologies, biochemical activity, and microbial profiles across individual plants, making each root system unique. Synthetic biology provides a potentially powerful approach to untangle and optimize the different processes that determine root system structure and function by establishing in vivo models where these relationships can be rigorously explored^2,3^.

Although we may not know all the consequences of modifying gene regulatory networks that control root system architecture, rhizodeposition, and root-microbe interactions, designing control over these processes is an important first step. This review first highlights plant genetic tools and tunable traits for designing roots with modified form, function, and rhizosphere interactions. Then, we discuss the process of engineering rhizobacteria as well as functions to target for improving crop performance in the face of climate change. From this exploration, we provide a holistic description of contexts in which a synthetic biology approach can be applied to plant root and microbiome engineering for improving crop resilience and sustainability.

### Engineering form and function of roots

#### Engineering predictable patterns of gene expression in roots

Most projects aimed at engineering the form and function of root systems will likely begin with plans to control the expression of genes that affect the development and physiology of specific tissues or root types^4,5^. Modifying gene expression in plants using characterized promoters is commonplace and is facilitated by sourcing and testing promoter sequences with published empirical evidence of activity^6,7^. Previous studies have utilized tissue-specific expression datasets generated by Fluorescence Activated Cell Sorting (FACS), and more recently single-cell RNAseq, to identify source promoters from genes expressed with patterns of interest (e.g., root tissue-specific). While the use of such curated promoter parts (DNA sequences with defined functions that can be used as modules in a synthetic gene) is often sufficient to create reporter genes, the design specifications required to engineer a plant with a specific form/function often require much finer control of gene expression programs at both the spatial and magnitudinal level^4^. Recent work has expanded the tools available for designing specific patterns of gene expression by utilizing synthetic transcription factor-based and DNA recombination-based circuits.

**Fig. 1:**
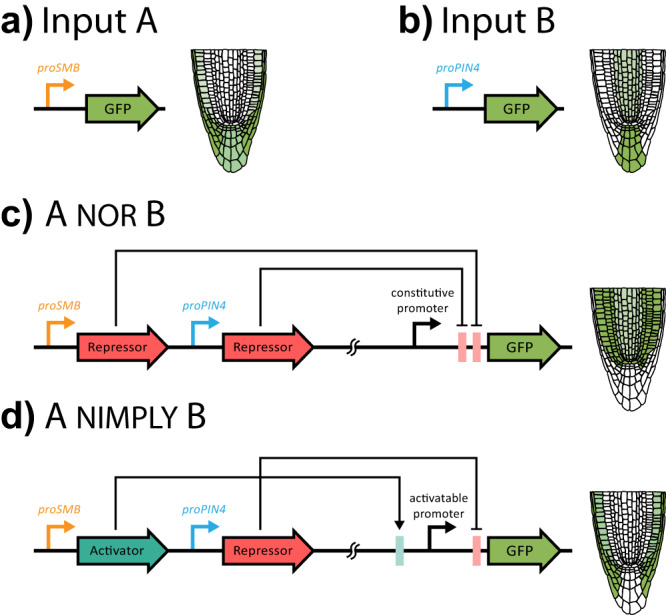
Two-input logic gates controlling tissue-specific gene expression in roots. Native promoters such as the **a** *SMB* promoter (columella and lateral root cap) and **b** *PIN4* promoter (columella and stele) can be used to drive tissue-specific expression of genes. By combining these promoters with synthetic activators and repressors, Brophy et al. generated circuits that can perform Boolean logic operations, creating novel patterns of gene expression not found in nature. **c** In the NOR gate, GFP is expressed only in tissues where both the *PIN4* and *SMB* promoter are not active. **d** In the NIMPLY gate, GFP is expressed only where *SMB* is active, but *PIN4* is not.

**Fig. 2:**
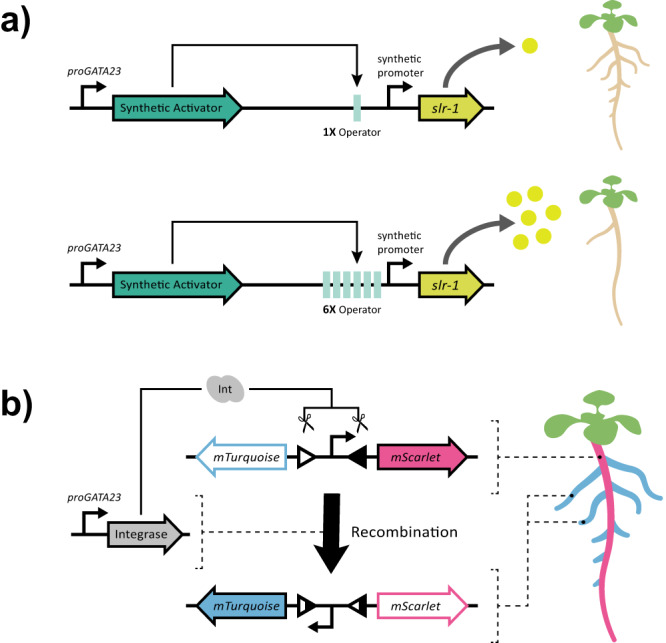
Controlling expression through buffer gates and recombinase-based circuits. Successful engineering of plant form will require fine control over gene expression, both in terms of magnitude and spatial patterning. **a** Circuits implementing buffer gates to tune expression of *slr-1* (inhibits lateral root formation), allowed Brophy et al. to control the number of lateral roots formed. **b** Guiziou et al. utilized a serine integrase to create a circuit that permanently switches its output when the integrase is expressed, creating a form of cellular memory that records past gene activity.

In Brophy et al., a large collection of sequences encoding DNA binding domains, sourced from various bacterial species^8^, was used to create synthetic transcription factors able to activate or repress expression of target genes in plants^9,10^. The well characterized DNA-binding specificity of these domains facilitated the construction of synthetic promoters responsive to the activity of the synthetic transcription factors. Screening this collection of transcription factors led to the identification of a subset that showed little cross reactivity, meaning they regulated expression predominantly through synthetic promoters that contained their cognate recognition sequence and not that of the other synthetic transcription factors tested (so-called orthogonality). The orthogonal nature of these synthetic transcription factor-promoter pairs facilitated the assembly of gene circuits capable of performing 14 different 2-input Boolean logic functions in tobacco transient assays. Boolean logic functions define the relationship between the activity state of inputs for the circuit and the circuit output. For example, an AND logic gate restricts the activity of the circuit to situations where both inputs to the circuit are in an ON state while activity is OFF when one or neither input is active. Successful circuit architectures were then ported into Arabidopsis, driven by native tissue-specific promoters, to create novel expression patterns based on the computed logic conferred by the specific circuit architecture. This system allows for the control of both the spatial pattern (Fig. 1) and expression level (Fig. 2a) of a downstream gene and utilizes a collection of parts with little sequence similarity, which limits the likelihood of gene silencing due to the presence of repetitive elements^11^.

In Lloyd et al., sequence-specific DNA recombinases were used, as an alternative approach, to trigger a stable change in synthetic genetic circuit architecture to permit or block the expression of a downstream reporter gene^12^. This system enables stable changes in gene expression since the circuit can be irreversibly modified by the recombinase. This irreversible nature may also allow the circuits to be especially sensitive to the expression level of the recombinase when traditional reporters prove insufficiently responsive. However, spurious activity of the synthetic gene circuit could occur if the promoter exhibits low-levels of expression outside of the domain of interest.

More recently, Guiziou et al. has demonstrated the utility of recombinases to record gene activity during lateral root development^13^. Here, serine integrases were used to flip a promoter element from a state that drives the expression of mTurquoise to an alternative state driving mScarlet expression (Fig. 2b). Input promoters were used to drive the expression of a serine integrase in the pericycle, which is a subpopulation of cells in the root that contributes founder cells for lateral root development. Successful implementation of the circuit led to mScarlet expression exclusively in lateral roots. In addition, combinatorial logic was introduced into these circuits by splitting the integrase coding sequence into two parts and fusing each half with the N or C-terminal half of an intein coding sequence (a splicing protein)^14^. Expression of the fusion proteins in the same cell allows for reconstitution of the integrase and recombination of the downstream reporter. Such combinatorial logic would allow for the recording function of the circuit to be triggered at a time of interest, or allow for greater tissue or condition specificity.

Together, these recent advances in synthetic gene circuits provide greater opportunities for researchers to tailor their synthetic gene architecture to the design specification of the target pathway. Transcription factor-based circuits enable analog or graded patterns of input promoter activity to be translated into graded patterns of circuit output, while recombinase-based systems generate digital (on/off) circuit outcomes that are stable over the long-term. Combination of these circuit modalities may allow for a wider spectrum of gene regulatory patterns to be engineered^4^.

#### Root architecture for water and nutrient uptake

**Fig. 3:**
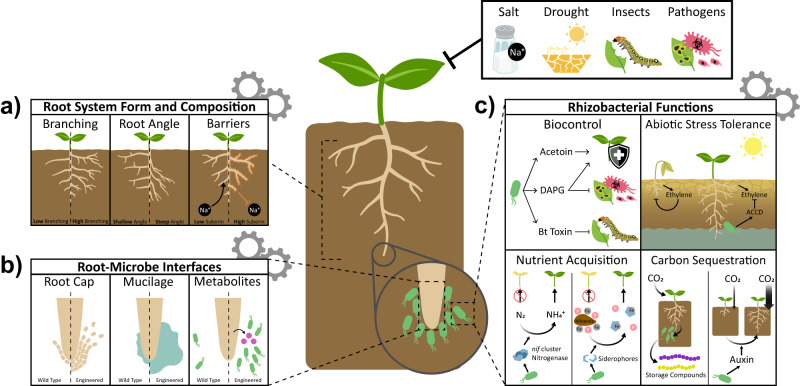
Tuning the rhizosphere through root and microbial engineering. Future climate conditions will exacerbate abiotic (salt, drought, etc.) and biotic (pathogens and pests) stressors that negatively impact crop yield. Through synthetic biology, root form, function, and microbial interactions can be altered to create new crops better equipped to grow in these more challenging conditions. **a** Custom root system architectures can be created by changing branch rate and gravity setpoint angle, resulting in root systems more suited for water and nutrient acquisition. Modulating suberin deposition can limit the uptake of toxic sodium and metal ions, while insulating roots against nutrient loss. Each panel represents a trait to target for engineering. Left of the dashed line are roots resulting from decreasing the target trait. Right of the dashed line represents an increasing target trait. **b** Primary and lateral root apices are the main interfaces at which plants modify the local soil environment, and by extension the composition of their microbiome, through the process of rhizodeposition. Control over root cap shedding dynamics and mucilage release can potentially improve root penetration into soil and drought resistance. These features, as well as the release of certain sugars and other metabolites, are also attractive engineering targets for controlling the composition of the root microbiome. Each panel represents a trait to target for engineering. Left of the dashed line is the wild type condition. Right of the dashed line depicts an engineered root. **c** The plant root microbiome expands the genetic repertoire available to the plant, providing a plethora of beneficial functions to their host. The metabolic flexibility of bacteria allows for the potential engineering of a myriad of actuators to improve plant biotic and abiotic stress tolerance, nutrient acquisition, and carbon sequestration.

Root systems in flowering plants are hierarchically branched structures^1,15^. Computational modeling of root system growth and physiology has revealed design rules that provide target goals for engineering^16–19^. In particular, work by the lab of Jonathan Lynch has established the steep, cheap, and deep ideotype for drought and nitrogen efficient agriculture^20^. This work has been heavily reviewed, but two traits (root system branching rate and gravity setpoint angle) are worth exploring here as their genetic basis has been more clearly defined and the application of synthetic gene circuits to modulate these traits is on the horizon (Fig. 3a). Furthermore, anatomical features of roots play prominent roles in defining their physiological interactions with soil water and nutrients^21,22^, and will be reviewed as an additional potential target for engineering.

#### Root system size

Root system growth is heavily dependent on the formation of lateral root branches^1,23,24^, either from the primary root axis, or in the case of grasses and some eudicots, from branches that form from the base of the shoot^15,25^. In Brophy et al., the authors utilized synthetic gene circuits to tune the level of expression for a mutant signaling protein *solitary root* (*slr*)/*iaa14*–*1* (Fig. 2a)^10^, which dominantly inhibits the transcriptional response to the auxin hormone^26,27^. In Arabidopsis, expression of *slr/iaa14*–*1* from its native promoter causes a complete suppression of lateral root development as well as many other pleiotropic effects on shoot growth and root hair development^10,27^. To control these effects, expression of *slr/iaa14*–*1* was limited to the pericycle tissue of roots, where lateral root primordia are induced, using the promoter *proGATA23*^10^. Quantitative tuning of lateral root development was then achieved using a buffer gate circuit architecture (Fig. 2a) where modifying the synthetic promoter driving *slr/iaa14*–*1* expression affected its responsiveness to a synthetic transcription factor that was expressed from the *GATA23* promoter. Reducing the number of cis-elements in the synthetic promoter and mutating these binding sites to reduce affinity for the transcription factor were both required to express *slr/iaa14*–*1* at low enough levels to quantitatively affect lateral root number without blocking their development entirely. Similar approaches may be useful in grass species since dominant-negative AUX/IAA mutant genes have been shown to have similar effects on root branching^28^.

#### Modulating gravity setpoint angle

In addition to root system size, the placement of branches and their subsequent growth angle, with respect to gravity, can determine the relative efficiency that resources are captured from soil^20^. Root growth is guided by the response to gravity and the angle that root growth takes concerning the gravity vector is called the set point angle^29,30^. Lateral roots that grow more steeply will establish a deeper root system, while a more shallow orientation will increase exploration of soil closer to the surface. Several pathways have been identified that affect the angle of emergence and subsequent growth of lateral roots and some of this work has demonstrated adaptive value of genetic loci that promote deeper root systems under drought conditions in the field^31–33^. What is less clear is how effectively these pathways can be controlled to tune root system architecture. Loss of function in the newly discovered *EGT2*/*WEEP* genes leads to steeper branch angles in the roots of wheat, barley and Arabidopsis, suggesting a conserved function across flowering plants, making this an attractive pathway for engineering^34,35^. However, whether the expression level of these genes can affect gravity set point angle in a quantitative manner has not been tested. Overexpression of *AtDRO1* in the TAC/LAZY family causes lateral roots to exhibit somewhat steeper growth trajectories^33^, however, like *EGT2*/*WEEP*, the effect is not specific to roots and gravity responses of axillary shoots are also affected. Surprisingly, only a few promoters have been directly tested for their ability to control gene expression exclusively in root or shoot tissues^36–39^. Establishing synthetic gene circuits that limit gene expression to above or below ground organ systems will be necessary to implement this engineering strategy, though this will be especially challenging in grasses where the majority of root biomass forms from shoots. Furthermore, manipulation of gravity set point angle in specific root types will likely be important for targeted improvements in root architecture and to prevent competition between roots of the same parent plant.

#### Modifying the selective uptake of solutes from soil

In roots, the cell-type specific pattern of cell wall modification enzymes and their regulators promote the formation of apoplastic (extracellular) barriers such as suberin and lignin^21,40–42^, which limit the passive movement of water and solutes in the tissue spaces outside of cells^22,43^. Engineering plants for sustainable agriculture will likely involve modifying these transport-associated pathways to enhance uptake of limiting nutrients, or to limit the uptake of toxic solutes, such as sodium and metal ions—conditions that are more likely to be present in degraded or marginal agricultural lands. Current research suggests that suberin is the more plastic apoplastic barrier component and hormone signaling pathways converge on the regulation of suberin biosynthesis genes in endodermal cells^22^. Furthermore, research into the patterning of the exodermal cell layer, which contains similar cell wall modifications as the endodermis and performs functions associated with drought and flooding tolerance, is also being explored and may lead to the ability to engineer transferable traits to new cell layers^44^. A better understanding of the different mechanisms that operate in the root to mediate communication and resource exchange with the rhizosphere, and its associated microbes, may also help leverage key root microbiome members to support plant and soil health.

### Engineering the root-soil metabolic interface

Roots are a major soil interface for chemical exchange where diffusion, transport, new growth, and cell death render a revolving door of nutrient uptake and release. Across woody and herbaceous taxa, plants can deposit 10–90% of fixed carbon to the root system^45,46^. Thus, engineering plants to modulate the composition and quantity of these deposits may be a powerful approach to manipulating soil properties and a potential route for sequestering carbon below ground (Fig. 3b).

Hundreds of compounds are released from roots from long sugar polymers to central metabolites^47,48^. The mechanisms that regulate the production and spatial patterns of rhizodeposits are largely differentiated based on the molecular weight of their outputs. For the secretion of low molecular weight exudates, diffusive pores and active transporters vary in distribution and can localize to specific root regions, while mucilage, a hydrogel composed of a complex mixture of polymers and metabolites, is released by root cap tissues present at the root tip and linked to specific developmental stages of the tissue^49,50^. Studies suggest that the exudation profile of a plant root system is primarily determined by plant genotype. Natural variation in exudate quantity and profile is observed across species, cultivars, plant age, root types, root developmental zones, and growing environments^51–54^.

#### Root cap development and shedding dynamics

The root cap is a regenerative tissue that sheds cells into the soil to protect the root apical meristem. In most species, such as *Pisum sativum* (pea) and maize, root cap cells shed individually as living border cells, which survive in the soil for a brief period of time before undergoing programmed cell death^55^. In *Arabidopsis thaliana*, some other members of the *Brassicaceae* family (mustard, canola, and cabbage), and certain tree species, the entire outer root cap layer sheds as a single sheath of connected cells called border-like cells^56–59^. Given the range of root cap morphologies and developmental programs, engineering the size, shape, and nature of root cap clearance has the potential to affect root and root-microbe activity.

Rhizodeposition is largely mediated by non-dividing cells in the lateral root cap (LRC) and the columella. Lateral root cap cells undergo developmental programmed cell death (dPCD) and detach from the root via autolysis. In the columella, living cells are sloughed into the rhizosphere before undergoing dPCD^60^. These sacrificed root cap cells likely absorb mechanical shear as the roots drill through soil via new growth^61^. Thus, changes in root cap development could affect the capacity of the root to penetrate soil. In *Arabidopsis*, genetic regulation of root cap development has been partially deciphered. FEZ and SOMBRERO (SMB) are NAC-domain transcription factors and control the orientation of stem cell divisions and subsequent root cap daughter cell maturation, respectively. The *fez* mutant has fewer lateral root cap layers while the *smb* mutant produces an extra cell layer and has a root cap that extends past the meristematic zone. Because cells in the *smb* root cap mature more slowly, these plants also exhibit slower rates of root cap shedding. Consistently, mutations in these genes were found to impact penetration into gelled media^62^. To overcome soil density at greater depths, root cap engineering may need to be complemented with modifications of root hairs and lateral roots as penetration anchors, as well as changes to root angle, cell wall rigidity, and root diameter to ensure that new root architectures can be implemented in a range of soil types^63^.

#### Mucilage release

As a slimy matrix, mucilage provides lubrication to enable growing roots to penetrate the soil^61,64^. In contrast to small metabolites, root mucilage is highly hygroscopic and affects soil wetting properties^65^. Some major components of mucilage include pectin, cellulose, hemicellulose, and arabinogalactan proteins^48,53^. Roots modulate rhizodeposition according to environmental, pest, and nutrient signals. For example, mucilage release was observed to increase under moderate drought but decrease under severe drought^66^. Mucilage can vary in its chemical properties, which affects the way it interacts with ions present in the soil. Pectic mucilage in the halophyte (salt tolerant) *Kosteletzkya virginica* was found to sequester sodium ions across a range of tissues including the roots^67^. In a study of aluminum tolerance, mucilage from *Melastoma malabathricum* sequestered more cations than maize^68^.

Mucilage can serve as the sole carbon source for microbes and was found to stabilize the catalytic activity of soil microbes under drought conditions^69,70^. In maize, the mucilage of aerial brace roots supports colonization by nitrogen-fixing diazotrophs^71^. Thus, engineering roots to release different quantities or compositions of mucilage could be used to modify the physical, chemical, and biotic environment of the root. Currently, however, the few genes known to affect mucilage production and release also disrupts the development of the root cap, which performs many other physiological functions^57^. Further investigation into the downstream targets of root cap developmental regulators may uncover new genes for the engineering of this vital rhizodeposit.

#### Root metabolite release

Engineering exudation of root released metabolites is one strategy to promote the stable colonization of plants by commercial inoculants or attraction of natural beneficial bacteria^72^. In a study of *Pseudomonas fluorescens* root colonization, L-malate, but not D-malate, induced chemotaxis^73^. After confirming positive chemotaxis to a plant defense compound, 2,4-dihydroxy-7-methoxy-2H-1,4-benzoxazin-3(4H)-one (DIMBOA), Neal et al. observed lower levels of *Pseudomonas putida* on the roots of DIMBOA-deficient *bx1* maize mutants^74^. Identifying specific chemoattractants and repellants released by roots or tuning host metabolism and incorporating novel microbe metabolic pathways are ways to manipulate community profile and root attachment. Understanding the impact of microbial chemoeffectors on plant host activity is also crucial to engineering sustained interactions in the rhizosphere. D-galactose released by cucumber roots is a strong chemoattractant for *Bacillus velezensis* SQR9. Roots exuded more galactose in response to SQR9, and supplementing the media with galactose increased *B. velezensis* root colonization^75^. This type of positive feedback in rhizosphere interactions has important implications for engineering persistent interactions between plants and microbes.

Engineering plant exudates can also enhance responses to pests and pathogens. Root exudates from knockdown lines of the gene *ABC-C6* repelled two species of parasitic nematodes^76^. Supplementing roots with fractionated exudates determined that increased levels of hexadecaonic acid and pentadecane were the primary chemorepellants^77^. In a related study, root exudates from knockdown lines of the ethylene response genes *ERF-E2* and *ERF-E3* were more attractive to nematodes, while chemotaxis of *Bacillus subtilis* or *Agrobacterium tumefaciens* were not affected^78^. Warnock et al. applied exogenous dsRNAs to silence sugar transporters and observed reduced levels of exuded glucose, fructose, and sucrose, which inhibited chemotaxis of two nematode species^79^. The multifunctional nature of exuded metabolites highlights the potential power of synthetic circuits to spatiotemporally tune the activity of exudate transporters to reduce unwanted attractant activity while preserving the role these molecules play in supporting the broader microbiome.

Another approach in exudation engineering is to select for microbes that can use a rare substance as a sole carbon source. Opines are molecules rarely found outside of the crown gall tumors induced by *Agrobacterium tumefaciens* and the genes for opine catabolism are not widely distributed. In 1997, Savka and Farrand engineered opine-catabolism into *P. fluorescens* and observed higher densities of growth on transgenic opine-producing tobacco^80^. On *Lotus japonicus* roots engineered to produce opine, opine-catabolizing microbe species were more represented in the rhizosphere^81^. Engineering new rare carbon-source metabolism and exudation in roots could aid bioprospecting efforts to identify specialized root colonizing microbes capable of metabolizing these food sources.

Using rare compounds in rhizosphere engineering could also create orthogonal systems that reduce the effect of a complex plant-microbe metabolome. Rhizopines are inositol-derived molecules specially produced and catabolized by nitrogen-fixing rhizobia in root nodules. They are functionally similar to *Agrobacterium* opines^82^. In 2019, Geddes et al. observed luminescence from a rhizopine *lux* biosensor in rhizobia on inoculated *Medicago truncatula* and barley roots harboring a synthetic rhizopine biosynthesis pathway^83^. In the complex signaling environment of the rhizosphere, genetic circuitry using these rare compounds allows for unique inputs for targeted manipulation of bacterial activity. However, engineering non-endogenous metabolic pathways requires extensive knowledge of biosynthetic pathways of both the target and competing compounds and preferred/alternative substrates.

### Engineering the microbial side of the rhizosphere

While plant synthetic biology approaches may one day usher in a generation of designer crops, the technical difficulties of plant transformation and genetic circuit design still limit the usability of these technologies outside of model plants. Bacteria, in comparison, benefit from fast generation times, predictive biophysical models, and broad host range techniques that have made rational design of genetic circuits possible, even in non-model species. The basic tools needed to deploy regulated circuits with functional outputs in plant beneficial rhizobacteria already exist, and may soon enable researchers to modulate plant function through their microbiome (Fig. 3c).

#### Designing and testing synthetic circuits in rhizobacteria

The successful function of a synthetic circuit depends heavily on its host chassis: the organism that houses and supports the designed circuitry. An ideal rhizobacterial chassis will be able to both accommodate heterologous protein/metabolite synthesis and robustly colonize the root. Root microbiome studies have identified a plethora of strains which both strongly colonize roots or have genetically tractable plant beneficial traits that can be mined for parts to use in engineering. Of these, root colonizing *Pseudomonas* spp. and *Bacillus* spp. are the most well studied and can be used to host a wide range of biosynthetic pathways. Alternatively, bacteria that are poor colonizers but possess other desirable features could be engineered for increased rhizosphere competence by altering traits such as biofilm formation, plant immunity evasion, and utilization of root exuded carbon^84^. The greatest challenge for any root colonizer will generally be competition with native soil microbiota which tend to quickly outcompete introduced strains, especially those hosting metabolically-costly machinery^85^. One solution is to use antibiotic producing chassis or engineering the expression of other biocontrol traits, in effect introducing a keystone species that carves out an ecological niche that impacts the rest of the microbiome. However, root association and community interactions are both complex polygenic traits and the underlying mechanisms for competitive colonization are not clearly understood, making engineering these features a non-trivial task. As an alternative to rational design, adaptive evolution can be used to generate strains with improved colonization in the lab. This technique has already been applied to evolve *Pseudomonas* and *Bacillus* strains with augmented colonization capabilities^86,87^.

The strength and expression pattern of different promoters and ribosome binding sites (RBS) can exhibit significant variability even between closely related strains^88^. Therefore, it is crucial to fine-tune these regulatory elements within a circuit to ensure functionality of its constituent genetic parts outside their original host contexts. Once designed, a genetic circuit must be empirically verified for activity in the chosen chassis, and will usually require optimization through multiple design-build-test-learn cycles.

While circuit designs are generally first assembled into self-replicating plasmid backbones, long-term functioning circuits are usually integrated into the genome of the target chassis to improve stability and remove the need for antibiotic selection. For precision engineering, CRISPR/Cas9 and its variants allow for specific editing of almost any site with a PAM sequence in a genome. Other technologies utilize modified ICE*Bs1* integrative and conjugative elements^89^ or recombineering^90^ to enable rapid genome integration of circuitry into a broad range of host strains.

#### Biotic stress tolerance

First identified in disease suppressive soils, rhizobacterial biocontrol strains that protect crops from insect pests and fungal/bacterial diseases offer attractive alternatives to environmentally damaging chemical pesticides. These strains can mediate plant resistance to pests and pathogens by modulating host immunity through induced systemic resistance (ISR), and/or by outcompeting, killing, or modulating attacking organisms. ISR protects plants against both disease and herbivory without major fitness costs^91^, but the mechanisms underlying its activation by rhizobacteria are not completely known and likely differ between plant/bacteria pairs. ISR activation in field plants is not ubiquitous, and requires ISR elicitor producing strains to pass a certain population threshold^92^. Thus, the ability to produce strong ISR elicitors at effective concentrations for host plants represents a prime target for engineering. A growing list of ISR elicitors, such as DAPG, 2,3-butanediol, and acetoin, have already enabled researchers to successfully engineer rhizobacteria with altered ISR activation capabilities^93^.

Better understood is the ability of certain biocontrol strains, in particular those of model biocontrol *Pseudomonas* and *Bacillus* species, to attenuate bacterial and fungal pathogens or insect herbivores. *Pseudomonas* spp. have been shown to protect a variety of plants by killing/inhibiting soil pathogens through the production of antibiotics (phenazines, pyoluteorin, pyrrolnitrin, 2,4-diacetylphloroglucinol/DAPG, etc), iron-chelating siderophores (pyoverdine and pyochelin), hydrogen cyanide, and toxin proteins^94,95^. Likewise, *Bacillus* spp. are known to produce iron siderophores and an array of antimicrobial/antifungal bacteriocins, lipopeptides, and polyketides^96^. *Bacillus thuringiensis*, which produces insect species specific Cry/Cyt toxin proteins, is the most widely used insect biocontrol strain and is already used in agricultural contexts worldwide for pest management. Outside of direct killing/inhibition, other rhizobacteria can modulate pathogen virulence via quorum quenching: the degradation of bacterial pheromones that mediate quorum sensing^97^. Using genome mining and comparative genomics tools, bacterial genomes can be analyzed to identify the genes and biosynthetic gene clusters underlying biocontrol effector synthesis^98^. Metabolic engineering can then be used to overproduce these targets in native contexts or port them to new chassis.

#### Abiotic stress tolerance

As climate change accelerates, crops will face abiotic stressors such as drought, high salinity, and extreme temperature with increasing severity and frequency. With this in mind, researchers have identified a plethora of rhizobacteria that confer abiotic stress tolerance to their host plants, either directly via the modification of the environment around the root, or indirectly by modulating host signaling and chemistry^99^. Rhizobacteria can alter soil chemistry around roots through the production of exopolysaccharide (EPS) matrices during biofilm formation, which increases water retention, improves soil macroporosity and adherence to the root, and restricts root uptake of Na^+^ ions and heavy metals^100,101^. Indirect mechanisms of increasing plant stress resistance involve modulating root architecture, growth inhibition, osmolyte accumulation, and ROS production in the host through the production/degradation of plant hormones (auxin/indole-3-acetic acid (IAA), 1-aminocyclopropane-1-carboxylic acid (ACC), cytokinins, abscisic acid (ABA))^102^, stress signals (trehalose, cadaverine)^103,104^, and other secondary metabolites (phenazine, 2,3-butanediol, etc.)^105^. It should be noted that the highly polygenic and pleiotropic nature of these resilience phenotypes can make it difficult to pinpoint specific mechanisms for engineering, though genetic determinants can still be identified in some cases. For example, several groups have altered EPS production and found that it is critical for the protective activity of *Bacillus amyloliquefaciens*, but whether EPS does so by altering soil chemistry, influencing plant stress responses, changing the colonization ability of rhizobacteria, or a combination of these factors is still unclear^100,101^. For the stress tolerance genetic actuators that have been identified, circuits for bacterial overexpression/heterologous expression of trehalose^106^, ACC deaminase^107,108^, and cytokinin^109^ successfully improved drought resistance in colonized plants. Using modern synthetic biology approaches, these circuits might be improved by adding the capability to sense and respond to environmental or host-derived stress signals or produce multiple synergistic tolerance actuators such as trehalose and ACC deaminase^110^.

#### Nutrient acquisition

Synthesis of ammonia for nitrogen fertilizer is energy intensive and generates significant greenhouse gas emissions^111^, making biological nitrogen fixation an attractive engineering target for improving the sustainability of non-legume crops. Minimal gene sets of the nitrogen fixation cluster (*nif*) are characterized for some species, but the complexity of nitrogenase assembly, the O_2_ sensitivity of its catalytic center, and relatively high fitness cost of hosting the pathway makes transfer of *nif* activity into non-native chassis difficult^112^. Engineering efforts have focused on optimization of protein stoichiometries^113^, reducing O_2_ sensitivity^114^, and altering downstream ammonia utilization/repression^112,115^, but have so far been unable to reconstitute native levels of *nif* activity.

Outside of N_2_ fixation, rhizobacteria can also improve acquisition of iron and phosphate, which are generally present in soil but not in bioavailable forms. Production of organic acids^116^, redox-active antibiotics^117^, and iron siderophores^118^ by rhizobacteria release and mobilize iron and phosphate ions from mineral complexes for uptake by plants. Rhizobacteria engineered to express heterologous organic acid operons demonstrated improved phosphate solubilization and promoted growth in rice^119^. Although heterologous siderophore expression has not yet been demonstrated in a rhizobacterial context, successful biosynthesis in other model gram-negative chassis suggests these same circuits can be applied to improve rhizosphere nutrient availability. In soil, a significant proportion of phosphate can also be bound in phytate, a plant-produced phosphate-storage molecule. Soil phytate is adsorbed to minerals, further decreasing phosphate bioavailability. Shulse et al. expressed 82 diverse phytases in 3 rhizobacterial chassis and identified 12 combinations that improved Arabidopsis growth via phosphate release from phytate^120^.

#### Carbon sequestration

Increasing the amount of carbon sequestered in soil can potentially offset a significant amount of anthropogenic carbon emissions, with the added benefit of improving soil quality^121^. Rhizobacteria are key players in this process, increasing soil organic matter (SOM) both directly through biomass accumulation and indirectly by influencing plant root growth^122^. A recent analysis by Tao et al. suggested that tipping microbial carbon usage to favor growth and byproduct formation over respiration is likely the most impactful strategy for increasing soil carbon sequestration^123^. This could be accomplished by using genetic circuits that increase the production of carbon rich storage compounds, such as polyhydroxyalkanoates, triacylglycerols, wax esters, and glycerol, or structural compounds such as bacterial cellulose^124^. An alternative strategy may involve increasing the amount of highly stable mineral-associated-organic-carbon in soil by elevating production of nitrogen rich carbon compounds that readily adsorb onto free mineral surfaces^125^. To avoid high fitness costs, any circuit will likely need to be regulated in response to carbon availability, so that synthesis of these compounds occurs only when excess carbon is available. In addition to storing carbon themselves, rhizobacteria can elevate plant carbon inputs to soil by altering root system size and architecture. Auxin/IAA producing rhizobacteria can increase primary root length and lateral root formation in plants^126,127^ and ACC deaminase producers can encourage root growth even under high stress conditions^128^, resulting in greater carbon input into soil as cellulose and other compounds.

#### Microbiome community engineering

Unreliable performance and stability in field conditions is the primary obstacle holding back beneficial rhizobacteria as a real-world solution for sustainable agriculture. While improving root colonization of individual strains can ameliorate this issue, the fact remains that environmental conditions and soil microbial consortia vary greatly from field to field^129^. To address this challenge, the next stage of rhizobacterial engineering will likely focus on microbial community engineering, controlling microbiome species composition or metagenome content to set the stage for optimal performance of synthetic circuits.

Approaches to community engineering can be generally split into bottom-up and top-down strategies. Bottom-up methods involve isolating and engineering individual strains to assemble into synthetic communities (SynComs)^130,131^. Simple SynComs have already demonstrated potential utility in the field, exhibiting greater stability and performance on plants compared to single-strain inoculations^132^. In contrast, the top-down approach aims to modify the metagenome of entire native communities, capturing as much natural diversity as possible while enhancing or removing target functions. Conjugation based tools XPORT^89^ and MAGIC^133^ utilize broad host range conjugative elements/vectors to first transform diverse species with engineered payloads and then utilize transposases to stably integrate circuits into genomes. Similarly, phages can be used to deliver and integrate circuitry into microbes. Although host range and payload size are more limited compared to conjugative tools, phages are well suited for targeting a single or small defined range of strains within a community^134^. Combining these tools with CRISPR payloads allows for gene level editing of the whole metagenome, potentially ‘deleting’ certain functions from an entire community^135^.

### Future perspectives and conclusion

While recent advances in the application of synthetic biology approaches to plants has established a foundation of tools for future studies, there are several areas where substantial bottlenecks to progress remain. Adapting circuits to crop plants is challenging since few characterized tissue-specific or environment specific promoters have been directly tested. While single-cell transcriptomics and assessment of chromatin accessibility are becoming readily applicable approaches in any species with a sequenced genome^136,137^, testing the activity of sourced promoters still requires the production of transgenic plants, which slows the initial stages of circuit design. Hairy root transformation systems may be useful in this regard. However, the physiological relevance of these systems may have significant limitations in certain contexts^138^. Transient transformation in *N. benthamiana* has served as an efficient means of prototyping circuit designs^10^, but due to phylogenetic distance, this approach may have more limited utility when engineering monocots. Use of transient transfection assays in grasses will likely facilitate this transferability gap^139^. Beyond engineering a single organism, engineering plant-microbe interactions will benefit from the development of rapid gene expression systems that facilitate genetic manipulation of both the plant and the microbe. *N. benthamiana* has served as a useful system for studying plant-pathogen interactions^140^, and it will be interesting to explore whether molecular pathways associated with commensal or symbiotic plant-microbe interactions can be modeled and engineered in transiently transformed leaf tissues. Arbuscular mycorrhizal fungi (AMF) represent another promising target for genetic engineering as they form symbiotic relationships with most crop plants. Stable genetic transformation of AMF has not yet been shown^141^, but once this roadblock is overcome, genetic parts and circuits tested in other fungal phyla could potentially be ported for immediate use in AMF, unlocking a treasure trove of new chemistry for improving agriculture. Currently, deployment of transgenic microbes in the field is not allowed and significant effort will need to be made to establish a regulatory framework that allows for innovation while also protecting the environment and natural biodiversity^142^.

Despite these current limitations, new tools for plant synthetic biology have empowered researchers with the fine spatial and magnitudinal control over gene expression needed to predictably alter form and function in plants. As our understanding grows, we may soon see the first designer root systems and microbiomes engineered to both survive climate change through increased stress resistance and fight it by augmenting soil carbon sequestration. The curtain is opening on the next stage of crop engineering, and it will be exciting to see the elaborate choreography that synthetic biologists create between plants and microbes to achieve sustainable agriculture in the face of climate change.

### Reporting summary

Further information on research design is available in the Nature Portfolio Reporting Summary linked to this article.

### Supplementary information

Reporting Summary
